# A simple and effective method to discern the true commercial Chinese cordyceps from counterfeits

**DOI:** 10.1038/s41598-020-59900-9

**Published:** 2020-02-19

**Authors:** Fu-Li Zhang, Xiao-Feng Yang, Dong Wang, Shao-Rong Lei, Ling-An Guo, Wen-Juan Liu, Jun Song

**Affiliations:** 1 Analysis and Determination Center, Sichuan Academy of Agricultural Sciences, Chengdu, 610066 China; 2 Institute of Quality Standard and Testing Technology Research, Sichuan Academy of Agricultural Sciences, Chengdu, 610066 China

**Keywords:** Target identification, Clinical microbiology

## Abstract

The Chinese cordyceps, a complex of the fungus *Ophiocordyceps sinensis* and its species-specific host insects, is also called “DongChongXiaCao” in Chinese. Habitat degradation in recent decades and excessive harvesting by humans has intensified its scarcity and increased the prices of natural populations. Some counterfeits are traded as natural Chinese cordyceps for profit, causing confusion in the marketplace. To promote the safe use of Chinese cordyceps and related products, a duplex PCR method for specifically identifying raw Chinese cordyceps and its primary products was successfully established. Chinese cordyceps could be precisely identified by detecting an internal transcribed spacer amplicon from *O. sinensis* and a cytochrome oxidase c subunit 1 amplicon from the host species, at a limit of detection as low as 32 pg. Eleven commercial samples were purchased and successfully tested to further verify that the developed duplex PCR method could be reliably used to identify Chinese cordyceps. It provides a new simple way to discern true commercial Chinese cordyceps from counterfeits in the marketplace. This is an important step toward achieving an authentication method for this Chinese medicine. The methodology and the developmental strategy can be used to authenticate other traditional Chinese medicinal materials.

## Introduction

The ascomycetes fungus *Ophiocordyceps sinensis* (anamorph: *Hirsutella sinensis*)^1^, which is synonymous with *Cordyceps sinensis* in the historical classification system^2^, colonises and mummifies the larvae of moth species in the family *Hepialidae* in the winter^1,2^. Then, during the next late spring or early summer, the multiplied fungi breaks the host’s exoskeleton at the head, forming a parasitic complex that comprises the remains of the caterpillar and a fungal sexual stroma bud^3–6^. This whole fruiting-body complex, with the characteristics “winter worm–summer grass”, is well known as Chinese cordyceps^7^ or “DongChongXiaCao” in Chinese^3,7–9^. It has a long history of being used for health and medicinal purposes in traditional Chinese medicine (TCM) as a tonic and renowned medicinal material^7–9^. Chinese cordyceps contains diverse bioactive ingredients^10,11^ and is widely used to enhance immunity or treat respiratory, bronchial and lung inflammation^1,6,12^.

Chinese cordyceps is distributed mainly in alpine regions at high altitudes(>3,000 m) on the Qinghai-Tibetan Plateau in southwestern China^13^. Owing to its strict host-specificity and confined geographic distribution^14,15^, the natural production of wild Chinese cordyceps is very limited. Habitat degradation in recent decades and excessive harvesting by humans have intensified its scarcity and increased the prices of natural populations^6^. Additionally, various counterfeit versions having a similar morphology, such as natural counterfeits *Cordyceps hawkesii* Gray (*C. gunni*), C*ordyceps*. *liangschanensis*, *Cordyceps. militaris*, *Cordyceps. barnesii*, *Cordyceps. gracilis*, and the root of the *Starchys geobombycis* plant, as well as artificial mimics made from dough, are traded as natural Chinese cordyceps for profit, thereby causing confusion in marketplace and affecting the safe use of Chinese cordyceps^16–20^. Furthermore, some fermented mycelial products from *O. sinensis* or *C. militaris* have been accidentally or intentionally labelled as “DongChongXiaCao”, which also causes confusion^16,18^.

The Chinese cordyceps is often used directly as a raw material, but it is also processed into a powder that is used in patented Chinese medicinal and health products, which makes it nearly impossible to identify based on morphological appearance^16^. To ensure the medicinal and edibility safety of Chinese cordyceps products in the marketplace, many detection methods for monitoring and verifying the Chinese cordyceps ingredients have been developed. The traditional methods, including macroscopic and microscopic observation^19,20^, which depend on the tester’s subjective judgment and professional experience, could effectively recognise the crude Chinese cordyceps, except as powdered products. Molecular biology techniques based on the internal transcribed spacer (ITS) sequence of Chinese cordyceps, such as DNA barcoding^17,21,22^, DNA barcoding coupled with high resolution melting^23^, loop-mediated isothermal amplification^24^ and nested PCR^25^, have been developed and used for species identification in recent years. However, none of these methods could clearly differentiate Chinese cordyceps from fermented mycelia in powdered form^16,17,25^. Because Chinese cordyceps is a parasitic complex, both the remains of the host larvae and the fungal sexual stroma are integral parts, as a commodity and raw medicinal material. Neither the fungus *O. sinensis* nor its host species are equivalent to the TCM Chinese cordyceps. Thus, a scheme that identifies both the *O. sinensis* fungus and its host species is theoretically reasonable and necessary, and it should more accurately identify Chinese cordyceps.

Duplex PCR technology is a good option for this purpose, because it can not only detect both targets simultaneously but is also simple and reliable. Minor genetic variations in *O. sinensis* from different geographical regions have been reported^26^, and there are up to 60 host species of the fungus *O. sinensis*^13,16,27^. Here, degenerate primer pairs were identified and designed, and a duplex PCR technique for Chinese cordyceps detection was developed. Additionally, it was confirmed that this strategy was applicable and could provide a new practical way to identify the TCM Chinese cordyceps.

## Results

### The development and optimisation of the duplex PCR method

**Figure 1:**
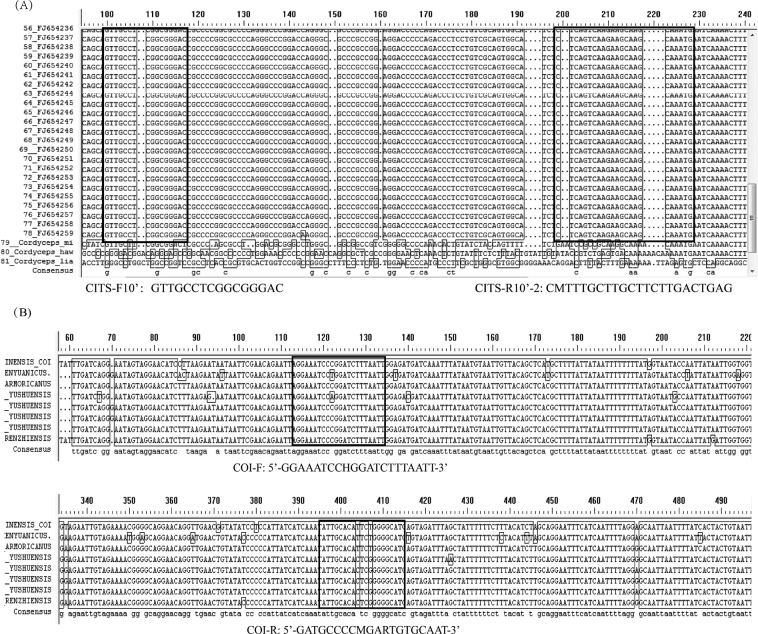
The sequence alignments used to design the primer pairs for Chinese cordyceps (DongChongXiaCao) detection. All the sequences were subjected to a BLAST search using DNAMAN software, and the black boxes indicate identical nucleotides. The nucleotide sequences of primers employed in this duplex PCR system are listed below each alignment. (**A**) Alignment of internal transcribed spacer sequences from *Ophiocordyceps sinensis* and its related species. In total, 78 ITS nucleotide sequences of *O. sinensis* and other related species taken from GenBank were aligned and compared. The positions of the CITS-F10′ and CITS -R10′-2 primers are indicated by bold black boxes. (**B**) Alignment of *COI* gene sequences from its host *Hepialiae* species. In total, eight nucleotide sequences of the *COI* gene were aligned and compared. The positions of the COI-F and COI -R primers are indicated by bold black boxes.

In total, 78 ITS nucleotide sequences of *O. sinensis* were aligned and showed high identity levels, and they differed from those of the counterfeit species (Fig. 1A). Areas that were conserved and stable among *O. sinensis* species but differed greatly in the sequences of other species were selected as targets for designing species-specific identification primers. Because a few base variation still existed in the conserved and stable regions within these species, degenerate oligonucleotide primers for the ITS region were designed for good compatibility. The same was true for the host species’ mitochondrial cytochrome oxidase c subunit 1 (*COI*) gene primers (Fig. 1B). Owing to the existence of single-base differences, degenerate primers were designed for the detection of the *COI* gene. Then, only 1 out of 17 primer pair combinations was selected for the detection of Chinese cordyceps. The CITS primer pair (CITS-F10′: 5′-GTTGCCTCGGCGGGAC-3′/CITS-R10′-2: 5′-CMTTTGCTTGCTTCTTGACTGAG-3′) and the *COI* primer pair (COI-F: 5′-GGAAATCCHGGATCTTTAATT-3′/COI-R: 5′-GATGCCCCMGARTGTGCAAT-3′), were further tested for their species-specificity and retained for subsequent experiments. The primer pairs, in same PCR reactions, at a 0.3-μM primer concentration and at an annealing temperature of 54 °C always generated clean band of the expected size for *O. sinensis* (113 bp) and its host *Hepialiae* (302 bp) in the positive control template. Then, the duplex PCR assay was successfully developed for Chinese cordyceps identification.

### Specificity of the duplex PCR

**Figure 2:**
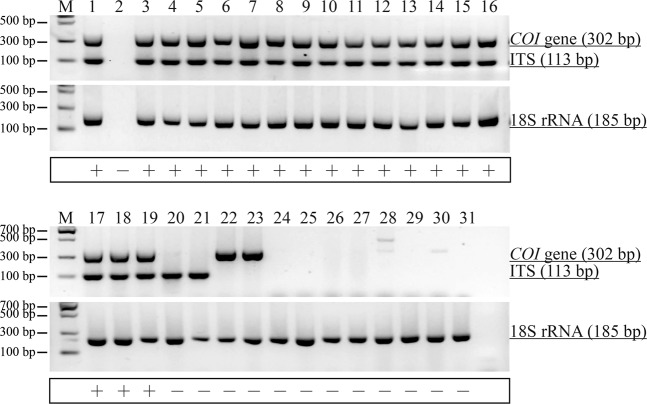
Specificity test of duplex PCR using primers specific for *Ophiocordyceps sinensis* and its host *Hepialiae* species. Lane M: DNA Marker II. Lane 1: positive control (No.121201); Lane 2: negative control (water); Lanes 3–19: raw Chinese cordyceps samples (1-QH-GL, 2-XZ-NQ, 3-XZ-LS, 4-XZ-LS, 5-XZ-SN, 6-XZ-LZ, 7-XZ-LZ, 8-XZ-CD, 9-XZ-LS, 10-QH-GL, 11-QH-YS, 12-QH-YS, 13-QH-HB, 14-QH-HN, 15-QH-TR, 16-SC-KD and 17-AB); Lanes 20 and 21: mycelial samples (23-JZ1 and 24-JZ2); Lane 22: moth (25-HY); Lane 23: XinJiangChongCao (22-XJCC); Lane 24: *Stachys geobombycis* (26-DC); Lane 25: *Euphorbia kansui* (27-GS); Lane 26: *Cordyceps militaris* (28-YCC); Lane 27: YuNanChongCao (18-YN); Lane 28: *Cordyceps gunni* (19-YXB); Lane 29: *Cordyceps liangschanensis* (21-LS); Lane 30: *Cordyceps sbarnesii* (20-XB); Lane 31: Mixed powder of several conventional crops (29-MC). The simultaneous appearance of the two target bands, one of the expected size for *O. sinensis* (113 bp) and one for the host Hepialiae species (302 bp), was considered a positive result and marked “+”. Others marked “−” were negative.

As shown in Fig. 2, the predicted 185-bp amplicon was amplified from all the samples tested, except for the negative control. Thus, DNAs over a wide taxonomic range can be detected, and the validity of the extracted DNAs for PCR can be assessed using the T18S-F/T18S-R primer pair. The amplicon could be used effectively as an analytical control to evaluate the authenticity of the template DNA.

Duplex PCR assays were further performed, and the expected fragments for the ITS (113 bp) and *COI* gene (302 bp) were successfully amplified from 17 Chinese cordyceps samples using the two primer pairs (CITS-F10′/CITS-R10′-2 and COI-F/COI-R), and two clear electrophoretic strips displayed the GelRed stain (Fig. 2). A single band of 113 bp appeared in the mycelial samples, and a band of 302 bp appeared in the moth and *C. gracilis* samples. No PCR products were detected from the other species tested, including the five readily confused varieties [*C. hawkesii* (*C. gunni*), *C*. *liangschanensis*, *C. barnesii* and *C*. *militaris*], the two counterfeit plants (*S. geobombycis* and *Euphorbia kansui*), one sample of a powdered crop mixture and the negative water control. The results showed that when the two target bands appeared from the same reaction, the test must be considered positive, and when none or only one target was detected, the test must be considered negative. In our specificity tests, two target amplicons were detected only in those samples identified preliminarily as Chinese cordyceps by the experts.

*Cordyceps gracilis*, named “XinJiangChongCao” in Chinese, is shaped like Chinese cordyceps. It is a complex containing the parasitic ergot fungus *C. gracilis* and its host lepidopteron larvae. The larvae of the moth *Hepialus altaicola*^28^, which is the host component of “XinJiangChongCao”, belongs to a species of *Hepialidae* family, which is the same as that of the host of Chinese cordyceps. As shown in Fig. 2, only the 302-bp host-specific amplicon appeared in the *C. gracilis* sample. This result adequately verified and confirmed the assay’s specificity.

A sequence analysis showed that the two amplified fragments were more than 99% identical to the corresponding *COI* gene of *Hepialidae* species. (GenBank Accession No: KM197522, KF696585, JQ325935, etc.) and the ITS region of *O. sinensis* (GenBank Accession No: KY321845~321856, MF598754, MF403011, KX082968, etc.), respectively. It was strong evidence for the specificity of the duplex PCR assay. Thus, the duplex PCR method developed in this study specifically identified the Chinese cordyceps.

### Sensitivity

**Figure 3:**
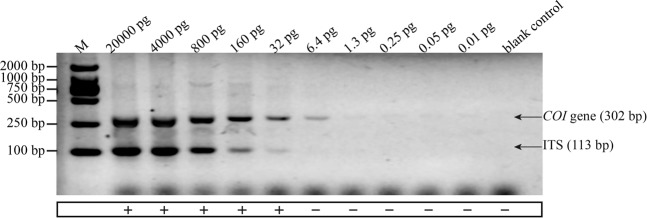
Detection limit of the duplex PCRs for both *Ophiocordyceps sinensis* and its host *Hepialiae* species. The template was genomic DNA extracted from reference material (No. 121201) and subjected to a fivefold serial dilution, resulting in approximately 20,000, 4,000, 800, 160, 32, 6.4, 1.3, 0.25 and 0.05 pg per PCR reaction. The DL2000 DNA Marker was used as the size scale for the target DNA. Sterile water was used as the negative blank control. The simultaneous appearance of the two target bands was considered a positive result and marked “+”. Others marked “−” were negative.

In the sensitivity test, 50.23 ng/μL total genomic DNA was initially extracted from the reference material, powdered Chinese cordyceps, and nine serial DNA dilutions were generated by fivefold serially diluting the initial extract. Then, 2 µL of each DNA dilution was added as the template in a PCR reaction, corresponding to approximately 20,000, 4,000, 800, 160, 32, 6.4, 1.3, 0.25 and 0.05 pg of the specific amplicon of the Chinese cordyceps per PCR reaction. As shown in Fig. 3, both target amplicons were simultaneously detected down to the 32 pg level in all the parallel reactions. The amplicons could not be detected in all the reactions at a lower template amount. Thus, the limit of detection (LOD), with a 95% confidence interval, for the duplex PCR assay was 32 pg in accordance with the criteria reported by Žel *et al*.^29^. Thus, the Chinese cordyceps was detected with an acceptable level of precision and accuracy. These results confirmed that the duplex PCR assay established in this study was a good approach for detecting and authenticating Chinese cordyceps.

### Applicability

Some commercial samples were tested. As shown in Table 1, in total nine raw samples (Samples 30-T, 32-T–38-T and 40-T) produced positive amplicons, and the target bands for the ITS from *O. sinensis* and the *COI* gene from the host *Hepialidae* species were synchronously amplified. No bands were detected in sample 31-T and three health-care products (Samples 42-T-44-T). Additionally, a single amplicon of *O. sinensis* was observed in sample 39-T and two other health-care products (Samples 41-T and 45-T). This confirmed that spurious products exist on the market and that the presence of Chinese cordyceps in the samples could be diagnosed accurately. The duplex PCR method developed in this study could distinguish products made from powdered mycelia of *O. sinensis* fungi from raw natural Chinese cordyceps. Thus, it is a quick and efficient way to identify and authenticate Chinese cordyceps.

**Table 1:** The test results for several commercial Chinese cordyceps samples.

Sample code	Sample definition	Target amplicons		Chinese cordyceps ingredient (Yes or No)
		ITS of *O. sinensis*	*COI* gene of *Hepialidae* species	
30-T	Chongcao	+	+	Yes
31-T	Chongcao	−	−	No
32-T	Chongcao	+	+	Yes
33-T	Chongcao	+	+	Yes
34-T	Chongcao	+	+	Yes
35-T	Chongcao	+	+	Yes
36-T	Chongcao	+	+	Yes
37-T	Chongcao	+	+	Yes
38-T	Chongcao	+	+	Yes
39-T	Chongcao	+	−	No
40-T	Chongcao	+	+	Yes
41-T	Corbrin Capsule	+	−	No
42-T	JinShuiBaoJiaoNang	−	−	No
43-T	Cordyceps pills	−	−	No
44-T	Cordyceps pills	−	−	No
45-T	Cordyceps MaiTake Capsule	+	−	No

## Conclusions and Discussions

Degenerate primer pairs targeting the ITS region of *O. sinensis* and the *COI* gene of its species-specific host were designed, and a duplex PCR assay for Chinese cordyceps was successfully established that can easily be performed in any molecular biology laboratory. It could specifically detect raw Chinese cordyceps and its primary components in products in which the genomes are undamaged. The results of the specificity analysis were in accordance with the preliminary identifications provided by experts. The LOD for Chinese cordyceps DNA could be as low as 32 pg. The accuracy and precision of the established duplex PCR method were confirmed and validated in this study. The applicability test further confirmed that the developed duplex PCR method can reliably identify and authenticate Chinese cordyceps. In addition, the duplex PCR method developed in this study is very simple to operate and cost-effective, which will be beneficial for its popularisation and utilisation. We believe that this method will be useful in the quality control and the identification of Chinese cordyceps and related products. The methodology and developmental strategy could be applied to other TCM materials as well, which will make authenticating TCMs easier. However, the method is not perfect. We were not able to differentiate mixed powders containing *O. sinensis* fungus mycelia and its insect host caterpillar from powders of the natural Chinese cordyceps using this method, although it was difficult to artificially raise the specific host insects required. In the future, chromatography methods that can detect the specific components of the Chinese cordyceps could be used together with the duplex PCR method. This may increase the reliability and accuracy of the identification of true Chinese cordyceps in any form.

## Materials and Methods

### Samples and materials

In total, 16 dried Chinese cordyceps samples (1-QH-GL, 2-XZ-NQ, 3-XZ-LS, 4-XZ-LS, 5-XZ-SN, 6-XZ-LZ, 7-XZ-LZ, 8-XZ-CD, 10-QH-GL, 11-QH-YS, 12-QH-YS, 13-QH-HB, 14-QH-HN, 15-QH-TR, 16-SC-KD and 17-AB) and one fresh Chinese cordyceps sample (9-XZ-LS) were collected from different sites in Qinghai, Tibet and Sichuan Provinces, where The Chinese cordyceps are mainly distributed. Four other varieties, *C*. hawkesii Gray (*C. gunni*) (19-YXB), *C*. *liangschanensis* Zang (21-LS), *C. barnesii Thwaites* (20-XB) and YunNanChongCao (18-YN) were collected in a local market in Qinghai. XinJiangChongCao [*C. gracilis* (Grev.) *duretMont*., 22-XJCC] was purchased from a local market in Urumqi, Xingjiang. The *C. militaris* (28-YCC) and two dried roots of *S. geobombycis* (26-DC) and *E. kansui* (27-GS) were purchased from a local market. A moth specimen (25-HY) of the family *Hepialiae* was provided by Harvard University. All the samples above were identified and confirmed by traditional morphological and DNA bar-coding method. A powdered reference material of Chinese cordyceps (No.121201) was purchased from National Institutes for Food and Drug Control, China. The mixed powder sample (29-MC) of conventional crop seeds including *Pisum sativum, Glycine max, Zea mays, Triticum aestivum, Oryza sativa* and *Vicia faba* were preserved and provided by Institute of Quality Standard and Testing Technology Research, Sichuan Academy of Agricultural Sciences. Two fungal strains (23-JZ1 and 24-JZ2) were isolated from stromal tissue of natural Chinese cordyceps specimens (9-XZ-LS) and were cultivated under previously described conditions^30^. The mycelia were then collected and conserved at −80 °C for extraction of genomic DNA. In total, 11 items (30-T~40-T), which were dried raw materials, labelled as Chinese cordyceps, were purchased as test sample from Jingdong Mall (an online shopping platform) (https://www.jd.com/) and Hehuachi Professional Market for Chinese Herbal Medicine, a local market in Chengdu. Two health-care products (41-T and 42-T) labelled with “DongChongXiaCao” were purchased from local TCM pharmacies in Chengdu, and three healthcare products (43-T–45-T) were purchased from Jingdong Mall. Detailed information regarding all the samples are shown in Table 2.

**Table 2:** Detailed information on the samples used in this study.

Sample code	Sample name	Brand	Description	Source	Acquiring way
1-QH-GL	DongChongXiaCao	—	Dried raw material	Guoluo, Qinghai	Collected by Dr. Youqing Bai
2-XZ-NQ	DongChongXiaCao	—	Dried raw material	Naqu, Tibet	
3-XZ-LS	DongChongXiaCao	—	Dried raw material	Lhasa, Tibet	
4-XZ-LS	DongChongXiaCao	—	Dried raw material	Lhasa, Tibet	
5-XZ-SN	DongChongXiaCao	—	Dried raw material	Shannan, Tibet	
6-XZ-LZ	DongChongXiaCao	—	Dried raw material	Linzhi, Tibet	
7-XZ-LZ	DongChongXiaCao	—	Dried raw material	Linzhi, Tibet	
8-XZ-CD	DongChongXiaCao	—	Dried raw material	Changdu, Tibet	
9-XZ-LS	DongChongXiaCao	—	Fresh	Lhasa, Tibet	
10-QH-GL	DongChongXiaCao	—	Dried raw material	Guoluo, Qinghai	Purchased from Sichuan Academy of Chinese Medicine Sciences
11-QH-YS	DongChongXiaCao	—	Dried raw material	Yushu, Qinghai	Collected by Mr. Ling Ma, a druggist.
12-QH-YS	DongChongXiaCao	—	Dried raw material	Yushu, Qinghai	
13-QH-HB	DongChongXiaCao	—	Dried raw material	Haibei, Qinghai	
14-QH-HN	DongChongXiaCao	—	Dried raw material	Hainan, Qinghai	
15-QH-TR	DongChongXiaCao	—	Dried raw material	Tongren, Qinghai	
16-SC-KD	DongChongXiaCao	—	Dried raw material	Kangding, Sichuan	
17-AB	DongChongXiaCao	—	Dried raw material	Aba, Sichuan	
18-YN	YuNanChongCao	—	Dried raw material	Lijiang, Yunnan	
19-YXB	YaXiangBangChongCao (*Cordyceps gunni*)	—	Dried raw material	unknown	
20-XB	XiangBangChongCao(*C. sbarnesii*)	—	Dried raw material	unknown	
21-LS	LiangShanChongCao(*C. liangschanensis*)	—	Dried raw material	Liangshan, Sichuan	Provided by Chengdu University of Traditional Chinese Medicine
22-XJCC	XinJiangChongCao(*C. grsacilis(Grev.) duretMont*)	—	Dried raw material	Altai region, Xinjiang	Purchased from local market in Urumqi, Xingjiang
23-JZ1	fungi strain	—	Fresh mycelium	—	Isolated from Fresh crude material 9-XZ-LS
24-JZ2	fungi strain	—	Fresh mycelium	—	Isolated from Fresh crude material 9-XZ-LS
25-HY	moth of the family *Hepialiae*	—	Dried raw material	Aba, Sichuan	Provided by Dr. Zhengyang Wang
26-DC	Dichan (*Stachys geobombycis*)	—	Dried raw material	unknown	Purchased from local market in Chengdu
27-GS	Ganshui (*Euphorbia kansui*)	—	Dried raw material	unknown	
28-YCC	YongChongCao (*C. militaris*)	—	Dried raw material	Chengdu, Sichuan	
29-MC	Mixed powder of several conventional crops	—	Dried raw material	Chengdu, Sichuan	Provided by Institute of Quality Standard and Testing Technology Research, Sichuan Academy of Agricultural Sciences.
30-T	Chongcao	Dongqiangtang	Dried raw material	Naqu, Tibet	Purchased from Jingdong Mall. (An online shopping platform)
31-T	Chongcao	Ayishe	Dried raw material	Naqu, Tibet	
32-T	Chongcao	Tongqinghetang	Dried raw material	Yushu,Qinghai	
33-T	Chongcao	Tongqinghetang	Dried raw material	Yushu,Qinghai	
34-T	Chongcao	Shanshijiagong	Dried raw material	Naqu, Tibet	
35-T	Chongcao	Zangzetang	Dried raw material	Naqu, Tibet	
36-T	Chongcao	Kaoshanzhang	Dried raw material	Naqu, Tibet	
37-T	Chongcao	Qingyuantang	Dried raw material	Yushu,Qinghai	
38-T	Chongcao	Yongzhiyuan	Dried raw material	Yushu,Qinghai	Purchased from local market in Chengdu
39-T	Chongcao	unlabeled	Dried raw material	unknown	
40-T	Chongcao	unlabeled	Dried raw material	unknown	
41-T	Corbrin Capsule	BaiLing	Processed health-care products	Hangzhou, Zhejiang	Purchased from Chinese herbal pharmacy in Chengdu.
42-T	JinShuiBaoJiaoNang	JinShuiBao	Processed health-care products	Yichun, Jiangxi	
43-T	Cordyceps pills	Kangfulai	Processed health-care products	Foshan, Guangdong	Purchased from Jingdong Mall. (An online shopping platform)
44-T	Cordyceps pills	Kangfulai	Processed health-care products	Foshan, Guangdong	
45-T	Cordyceps MaiTake Capsule	Mega	Processed health-care products	USA	
No.121201	The reference material of DongChongXiaCao	—	Powder	—	Purchased from National institutes for Food and Drug control, China.

### DNA extraction and purification

All the samples were treated and homogenised in liquid nitrogen. Genomic DNAs were isolated and purified from all the samples, except for two mycelia specimens, using a Plant Genomic DNA Kit (DP305-02, TIANGEN Biotech Co., Ltd., Beijing, China) in accordance with the manufacturer’s instructions. However, before that, the homogenised samples (50–100 mg each) were pre-processed in Proteinase K buffer [20-mM Tris-HCl (pH 8.0), 5-mM ethylenediaminetetraacetic acid (EDTA), 400-mM NaCl, 0.3% sodium dodecyl sulfate and 200 μg/mL Proteinase K (P6556, Sigma-Aldrich, Saint Louis, MO, USA)]. The resultant mixtures were incubated at 55 °C for 30 min, followed by 95 °C for 5 min. An equal volume of phenol/chloroform (1:1) was added to the suspensions and mixed fully for 5 min, and then, the mixtures were centrifuged at 12,000 × g for 5 min. The supernatants were transferred to fresh tubes and treated once with chloroform.

The total genomic DNAs of the two mycelia samples were prepared using the benzyl chloride method^31^.

The concentrations and qualities of the DNA samples were measured and evaluated using a NanoDrop ND-1000 UV/Vis Spectrophotometer (Thermo Scientific, Waltham, MA, USA) by examining the OD_260_/OD_280_ and OD2_60_/OD_230_.

### Primer design

ITS sequences from *O. sinensis* (GenBank Accession No: AJ243775, AJ243776, AJ243778, AJ245559, AJ309354, AJ309357, AJ309361, AJ413178–AJ413184, AJ507399–AJ507405, FJ654148, FJ654149 and FJ654206–FJ654259) and its related species *C. militaris* (GenBank Accession No: EU825996), *C. hawkesii* (GenBank Accession No:AJ536571), *Hirsutella* sp. (GenBank Accession No: EF469126) and *C. liangshanensis* (GenBank Accession No: AJ238503) were aligned using DNAMAN Version 6.0 (Lynnon Corporation, San Ramon, CA, USA) to select the species-specific ITS amplicons. Four kinds of Lepidoptera larvae, *Thitarodes*, *Ahamus*, *Hepialus* and *Parahepialus*, are the main host species of the fungus *O. sinensis*^27^. The sequences of *COI* genes from the host insects of *O. sinensis*, *Hepialus xiaojinensis* (GenBank Accession No: KP772242), *Hepialus menyuanicus* (GenBank Accession No: HM595858), *Hepialus armoricanus* (GenBank Accession No: HM595856), *Ahamus yushuensis* (GenBank Accession No: HM595854, HM595849, HM595840 and HM595850) and *Thitarodes renzhiensis* (GenBank Accession No: HM744694) were selected and aligned for species-specific amplicons of the *COI* gene. Owing to the minor variations that exist among the above sequences, degenerate primer pairs for the detection and identification of the commercial Chinese cordyceps were designed using Primer Premier Software (Version 5.0) (PREMIER Biosoft International, Palo Alto, CA, USA) and Primer Express Software Version 3.0 (Applied Biosystems, Foster City, CA, USA). They were synthesised by Sangon Biotech (Shanghai) Co. Ltd. (Shanghai, China). In addition, 18S ribosomal RNA gene sequences of *H. armoricanus*, and its closely related species and host plants (GenBank Accession No: JN036435, AF286273, KT343381, AJ830762, AJ830755, AF286298, KR068931, AJ830832, AJ830770, AJ830767, AJ830761, L37582 and U42535) were aligned. A specific universal primer pair (T18S-F: 5′-CGGAGAGGGAGCCTGAGAA-3′/T18S-R: 5′-GCACCAGACTTGCCCTCC-3′), producing a 185-bp amplicon, was designed and synthesised based on the highly conserved regions to validate the extracted DNAs for PCR.

### PCR conditions

Each PCR reaction was performed in a 25-μL reaction volume with 1× HotStar Taq^®^ Plus Master Mix (QIAGEN GmbH, Hilden, Germany), various concentrations of forward and reverse primers and 50 ng genomic DNA as the template. The tested primer concentrations were 0.16, 0.24, 0.32, 0.40 and 0.8 μM. Two primer pairs (CITS-F10′/CITS- R′-2 and COI-F/COI-R) were simultaneously added into the PCR reaction system to develop a duplex PCR assay for Chinese cordyceps identification. Primer combinations at different concentrations were tested to determine the optimal proportions that influenced the amplification efficiency. A gradient PCR was performed with annealing temperatures ranging from 50 °C to 60 °C in an ABI Veriti 96 thermal cycler (Applied Biosystems). Six temperatures in a gradient, 50 °C, 52 °C, 54 °C, 56 °C, 58 °C and 60 °C, were tested to determine the optimal annealing temperature. The program was as follows: one step of 5 min at 95 °C, 35 cycles of 30 s at 95 °C, 30 s at 50 °C–60 °C and 30 s at 72 °C; followed by one step of 7 min at 72 °C. The PCR products were size separated using electrophoresis on 2.5% agarose gel in 1 × Tris-acetate-EDTA buffer and stained with GelRed Nucleic Acid Stain (Cat. 41003, Biotium, Inc. Fremont, CA, USA) for visualisation. The amplicons were further confirmed by cloning and sequencing (Sangon Biotech (Shanghai) Co. Ltd.).

### Specificity test

To determine the specificity of the primer pairs and the obtained method, the duplex PCR was tested three times using both the *ITS* and *COI* gene primer mixtures and template DNAs from Chinese cordyceps samples (17), several easily confused varieties (5), counterfeit plants (2), mycelial samples (2), a moth from the family *Hepialiae* (1) and a powdered mixture of conventional crops seeds (1), as mentioned in Section 2.1.

Additionally, DNA from the Chinese cordyceps reference material was used as the positive control, and sterile water was used as the negative control.

### Sensitivity analysis

To further evaluate the sensitivity of the established method, genomic DNA from raw Chinese cordyceps was prepared and fivefold serially diluted using nuclease-free Tris-EDTA buffer to 0.05 pg/μL, and the gradient dilution technique previously published by Zhang *et al*.^32^. The duplex PCR assays were performed as described in Section 2.4, and the serial dilutions were used as the templates. All the reactions were repeated three times, with 15 parallel runs for each template DNA, to determine the LOD of the duplex PCR assay based on the positive result criteria^29^.

### Applicability test

To test the applicability of the duplex PCR method for analysing practical samples, 16 samples were purchased from a local market or Jingdong Mall, as described in Section 2.1. DNAs were isolated from 11 raw Chinese cordyceps samples and 5 health-care products containing Chinese cordyceps, according to the labels. Then, the duplex PCR assays were performed in triplicate using the obtained DNAs as templates. The genomic DNA isolated from the Chinese cordyceps reference material was used as the positive control. Sterile water was used as the negative control.
